# Endoscopic laser lithotripsy for an impacted pancreatic duct stone using a novel ultra-slim cholangiopancreatoscope via the minor papilla

**DOI:** 10.1055/a-2710-6280

**Published:** 2025-10-16

**Authors:** Takeshi Ogura, Junichi Nakamura, Takafumi Kanadani, Kimi Bessho, Hiroki Nishikawa

**Affiliations:** 1 38588 Pancreatobiliary Advanced Medical Center, Osaka Medical and Pharmaceutical University Hospital, Osaka, Japan; 2 38588 Endoscopy Center, Osaka Medical and Pharmaceutical University Hospital, Osaka, Japan; 3 13010 2nd Department of Internal Medicine, Osaka Medical and Pharmaceutical University, Osaka, Japan

An impacted pancreatic duct stone can be treated using several techniques, including endoscopic laser lithotripsy (ELL) under peroral pancreatoscopy 1 2 . However, if there is pancreatic duct dilatation upstream of a pancreatic duct stone, insertion of the pancreatoscope into the main pancreatic duct may be challenging. In addition, due to the limited space of the main pancreatic duct, ELL is sometimes challenging because it may be difficult to adjust the axis between the stone and the laser probe. To overcome this, an ultra-slim cholangiopancreatoscope with the unique characteristic of providing the working channel exit at the 3 o’clock position (7.8 Fr, Briview, SeeGen Co., Ltd., Shanghai, China) has become available ( Fig. 1 ). A case of successful pancreatic duct stone removal using this pancreatoscope is described.

**Fig. 1:**
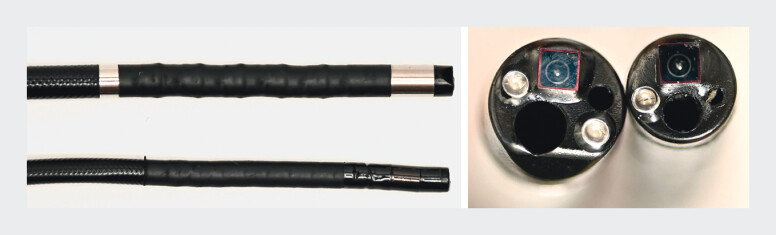
An ultra-slim cholangiopancreatoscope with the unique characteristic of providing the working channel exit at the 3 o’clock position (Briview, SeeGen Co., Ltd., Shanghai, China) (10, 2, and 7.8 Fr) has become available.

A 59-year-old man was admitted to our hospital due to pancreatitis caused by an impacted pancreatic duct stone. To remove the stone, endoscopic retrograde cholangiopancreatography (ERCP) was attempted. After successful pancreatic duct cannulation, contrast medium was injected, but the main pancreatic duct was too thin. Therefore, the guidewire was extracted from the minor papilla, and the duodenoscope was then pulled back to the minor papilla. Subsequently, minor pancreatic duct cannulation was successful ( Fig. 2 ). Although the guidewire was deployed across the pancreatic duct stone, the ERCP catheter could not be passed due to the impacted pancreatic duct stone. In addition, since the minor pancreatic duct was also not very dilated, insertion of the ultra-slim pancreatoscope was attempted. Scope insertion was smoothly performed, and the pancreatic duct stone was identified ( Fig. 3 ). If the ELL probe had been extracted from the 6 o’clock position, pancreatic duct injury might have occurred, whereas extraction from the 3 o’clock position allowed ELL to be performed without pancreatic duct injury ( Fig. 4 ). After successful ELL, impaction was resolved. Using a basket catheter, stone extraction was successfully performed without any adverse events ( Fig. 5 , Video 1 ).

**Fig. 2:**
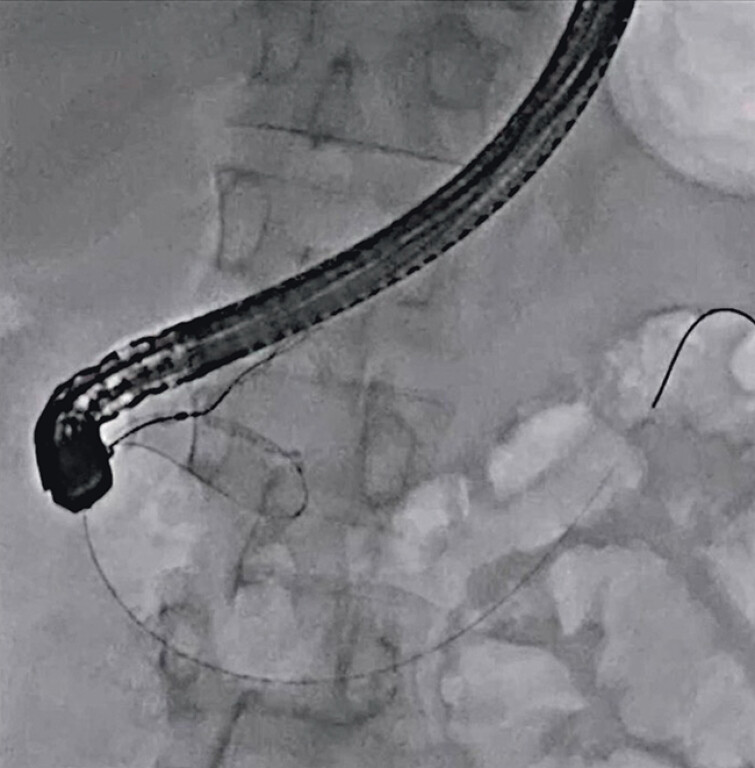
Minor pancreatic duct cannulation is successful.

**Fig. 3:**
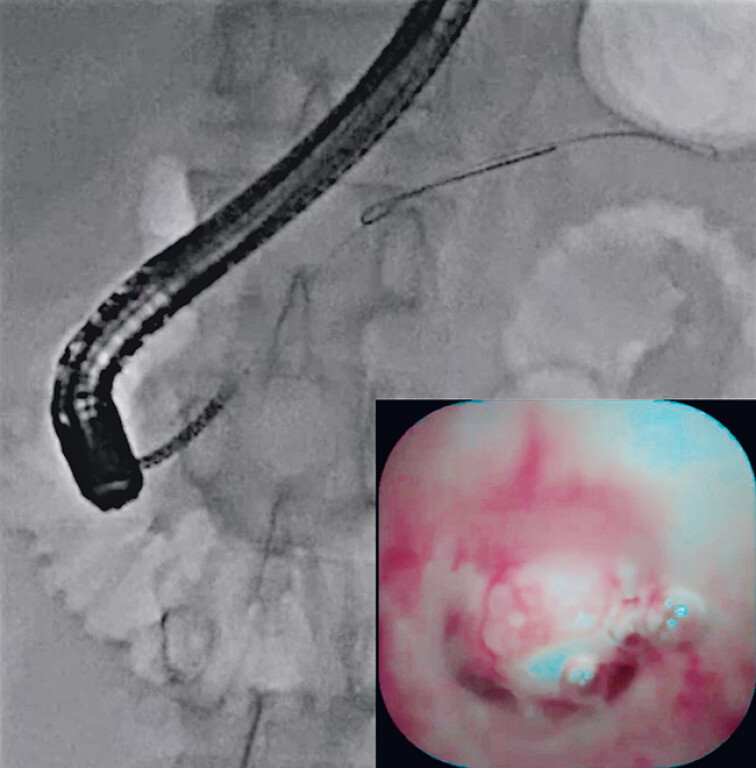
Insertion of the ultra-slim pancreatoscope is smoothly performed, and the pancreatic duct stone is identified.

**Fig. 4:**
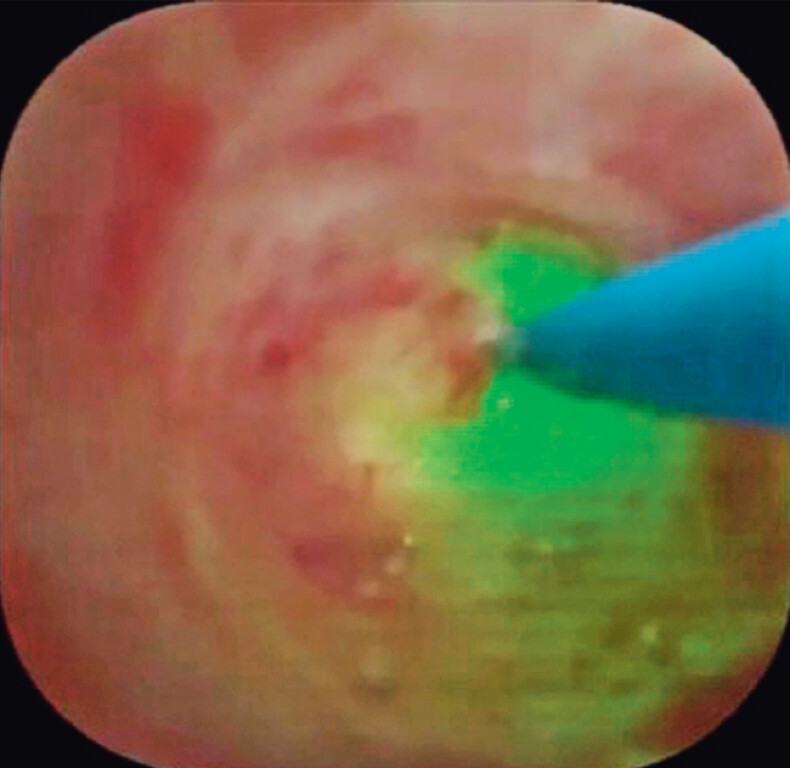
Extraction from the 3 o’clock position allows endoscopic laser lithotripsy to be performed without pancreatic duct injury.

**Fig. 5:**
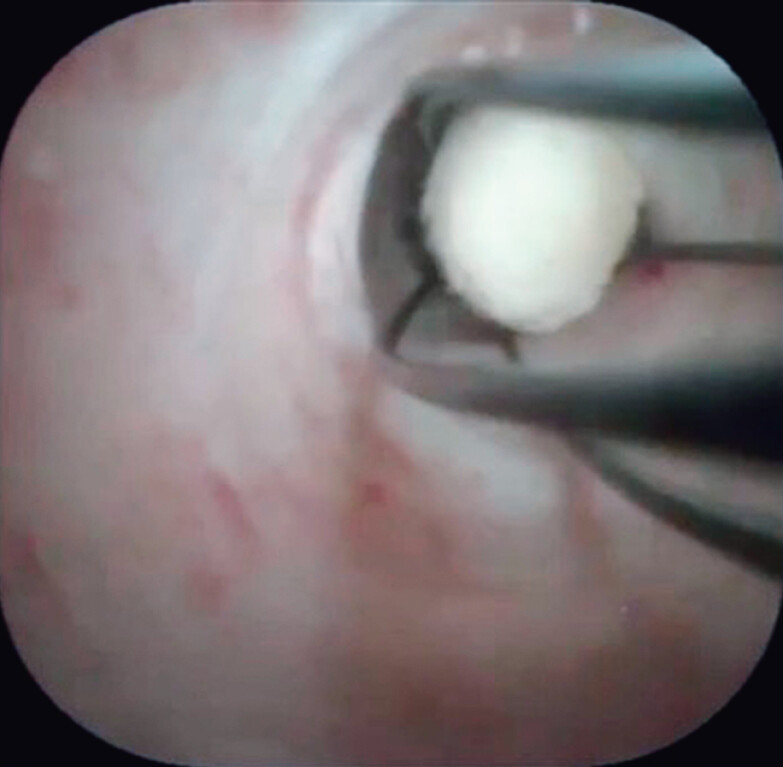
Stone extraction is successfully performed.

Endoscopic laser lithotripsy for an impacted pancreatic duct stone using a novel ultra-slim cholangiopancreatoscope via the minor papilla.Video 1

In conclusion, the novel cholangiopancreatoscope may be useful for performing safe ELL because of the working channel exit at the 3 o’clock position.

Endoscopy_UCTN_Code_TTT_1AS_2AI

## Notes

Endoscopy E-Videos https://eref.thieme.de/e-videos
*E-Videos* is an open access online section of the journal *Endoscopy* , reporting on interesting cases and new techniques in gastroenterological endoscopy. All papers include a high-quality video and are published with a Creative Commons CC-BY license. Endoscopy E-Videos qualify for HINARI discounts and waivers and eligibility is automatically checked during the submission process. We grant 100% waivers to articles whose corresponding authors are based in Group A countries and 50% waivers to those who are based in Group B countries as classified by Research4Life (see: https://www.research4life.org/access/eligibility/ ). This section has its own submission website at https://mc.manuscriptcentral.com/e-videos .
